# Case Report: Evidence of Migratory Silicone Particles Arising From Cohesive Silicone Breast Implants

**DOI:** 10.3389/fgwh.2022.730276

**Published:** 2022-04-25

**Authors:** Jessica C. R. Mustafá, Eduardo de Faria Castro Fleury, Henry B. P. M. Dijkman

**Affiliations:** ^1^UEMS Universidade Do Estado Do Mato Grosso Do Sul, Campo Grande, Brazil; ^2^Centro Universitário São Camilo, Curso de Medicina, São Paulo, Brazil; ^3^Institute of Applied Biosciences and Chemistry, HAN University of Applied Sciences, Nijmegen, Netherlands

**Keywords:** silicone breast implant, silicone gel bleed, silicone migration, histology, non-cohesive silicone gel, cohesive silicone gel

## Abstract

**Background:**

Silicone implants have been used since the 1960s for aesthetic purposes and breast reconstructions. During this period, many women have reported up to 40 similar symptoms, including fatigue, the emergence of autoimmune diseases, Raynaud Phenomenon, arthritis, arthralgias, and hair loss, among others. However, most of the time, these symptoms are neglected by doctors across different specialties and are most often considered a psychosomatic disease. Since 2017, many women suffering from the same complaints have formed social media groups to report their histories and subsequently describe the disease as Breast Implant Illness (BII). The phenomenon of gel bleed and silicone toxicity is known and accepted in literature, but silicone migration into the extracapsular space is still poorly demonstrated, due to the difficulty of monitoring its particles and access to patient data.

**Methods:**

This work demonstrated the presence of silicone through pathological examination in post-explant breast capsules and in the synovial tissue of the right wrist, detected with special Modified Oil Red O (MORO) staining in a patient with a history of BII. The pathological results were compared to the breast MRI imaging files.

**Results:**

The MRI images show the permeability change of the implant shell diagnosed as a water-droplet signal. It was also possible to diagnose the gel bleeding as the silicone-induced granuloma of breast implant capsule (SIGBIC) in both implants. Silicone gel bleed and migration of silicone were detected with MORO staining in and outside the capsule and in the synovial tissue of the right wrist.

**Conclusion:**

In this case study, we showed that silicone migration is possible *via* cohesive silicone gel breast implant leakage. The accumulation of silicone in the synovial tissue of the right wrist suggests local silicone toxicity and defects.

## Introduction

Silicone breast implants (SBIs) have been used since the 1960s for breast augmentation and reconstruction. Over the years, there have been great controversies about its biocompatibility and toxicity. Significantly, in 1995, Brautbar et al. proposed that silicones were not inert and could induce cell death and have mutagenic effects as a result of gel bleed and migration of silicone molecules (1–3). Some women with the most recent generation of cohesive SBIs have reported similar clinical complaints known as Breast Implant Illness (BII). BII is characterized by a set of 40 symptoms including, but not limited to, fatigue, arthralgia, autoimmune disease, and hair loss. However, these symptoms are often considered a psychosomatic disease. On 27 October, 2021 the FDA took several new actions to strengthen breast implant risk communication and issued a black box warning. Such warnings alert physicians and the general population that breast implants are not lifelong devices, there is an increased chance of complications (BII), and that SBIs have been associated with anaplastic large cell lymphoma (BIA-ALCL) and systemic symptoms (4).

This case report aimed to alert the medical community about the real migration of silicone particles to tissues, which is therefore associated with their deleterious cytotoxic effects.

Currently, there is a lack of standardization for BII diagnosis. No validated tests can infer silicone bleed and impact patient management. The diagnosis of silicone bleed is possible with special histological staining, such as Modified Oil Red O (MORO), associated with MRI or ultrasound to perform the diagnosis of gel bleed, rupture, or migration in the patient. We demonstrated that we can perform screening and diagnosis for patients suffering from BII.

## Materials and Methods

### Patient History

The patient involved has the initials JCRM, a 33-year-old woman with SBIs (Perthese Breast Implant 260cc) for aesthetic purposes in July 2005. After 6 months, nonspecific symptoms of fatigue, drowsiness, night sweats, emotional lability, Raynaud's Phenomenon, urticaria, and shortness of breath on exercise began, which were progressively aggravated over the course of 10 years.

In 2015, due to the symptoms worsening, immunological screening was performed, with a positive result in ANA 1/320 and anti-centromere 1/320.

During pregnancy in 2017, the patient presented marked edema of the upper extremities, resulting in severe compression associated with bilateral median nerve ischemia. JCRM underwent carpal tunnel surgery in the right wrist at 24 weeks of gestation followed by the left wrist at 28 weeks. Other events observed were mild proteinuria, arthralgias, and peripheral arthritis. The fetus showed intrauterine growth restriction, difficulty in fetal weight gain, and at-birth transient tachypnea.

Then, 3 months after delivery, pain with an inflammatory pattern in the sacral iliac basin began, which was associated with migratory peripheral arthritis. Treatment was performed *via* non-steroidal anti-inflammatory drugs (NSAIDs; naproxen and colchicine) but did not result in substantial improvement, and treatment with immunobiological, anti-interleukin 17 (IL-17) began in 2020. After 12 months of use, there was an improvement in symptomatology, but with a worsening of the ultrasound and MRI findings in the wrists. To improve the symptoms, the patient was submitted to microneurolysis surgery with a fat graft to the right median during which an incisional biopsy of the epineurium and synovia was performed. During the last year, the patient complained of slight tenderness on the left breast, which made her seek medical assistance. The complimentary ultrasound was negative for implant complications. She was referred to a diagnostic MRI.

**Figure 1:**
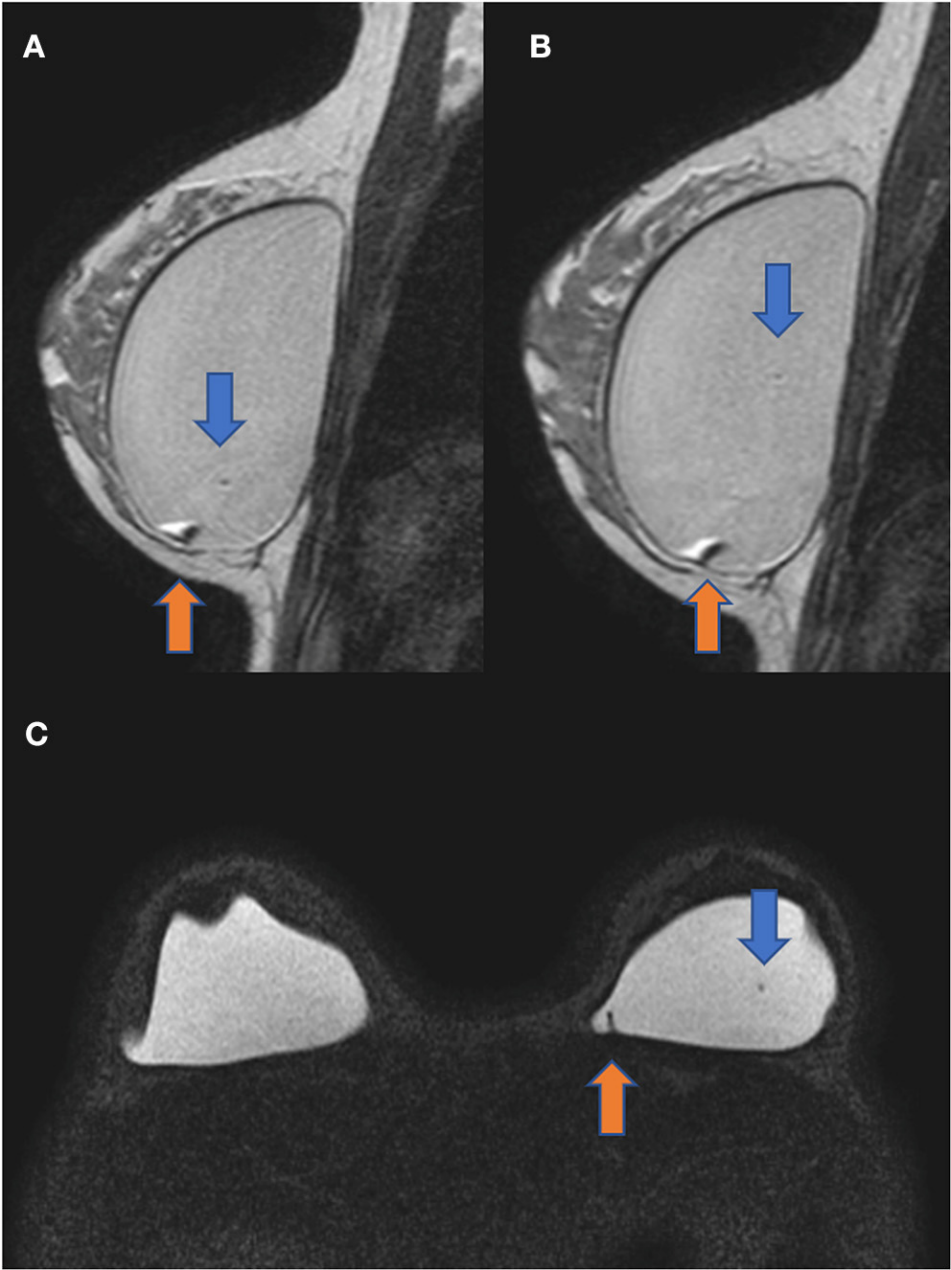
Sagittal proton density **(A,B)** and axial silicone only sequence **(C)**. The blue arrow shows the water-droplet signal inferring permeability change of the breast implant shell. The orange arrow demonstrates an intracapsular mass compatible with the silicone-induced granuloma of the breast implant fibrous capsule. There is also a capsular contraction in both implants.

**Figure 2:**
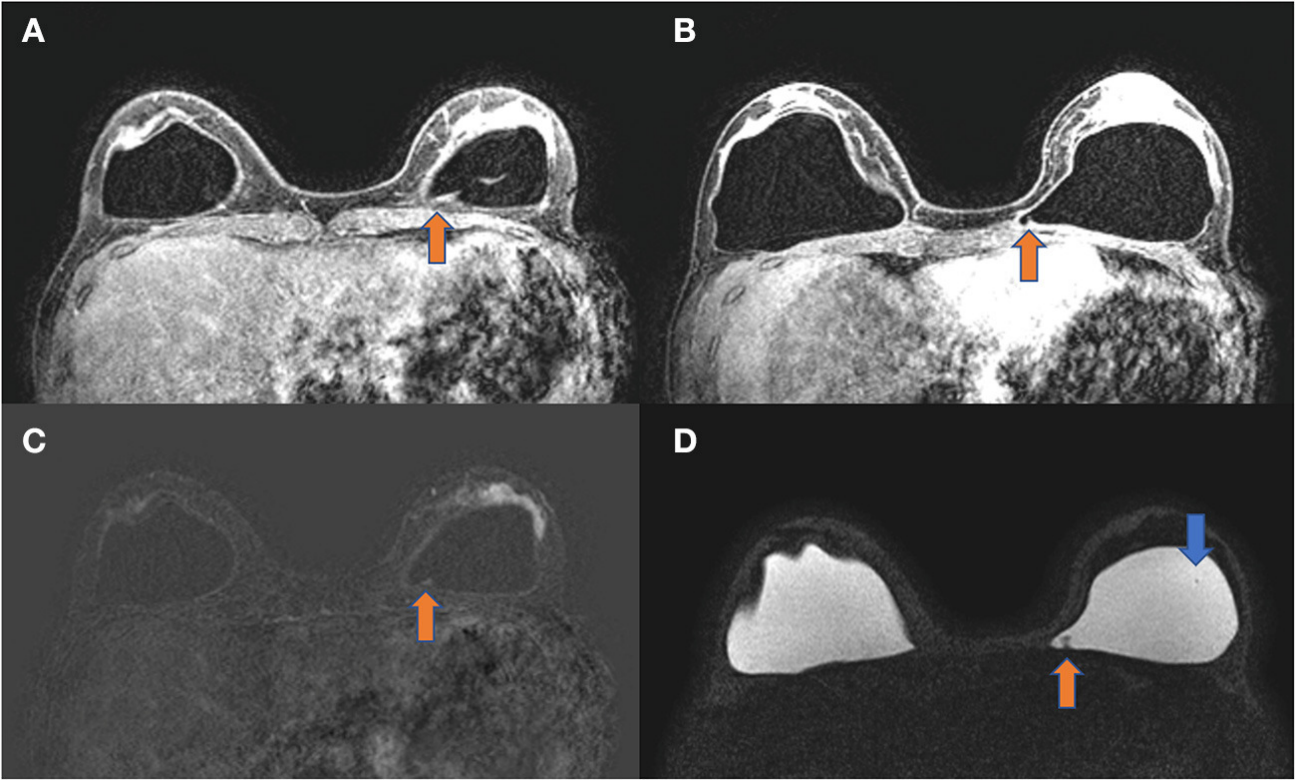
**(A–D)** Axial images of the same patient. T1 post-contrast **(A,B)**, T1 subtraction **(C)**, and silicone only sequence **(D)**. The orange arrow shows an intracapsular mass with late contrast enhancement compatible with silicone-induced granuloma of breast implant capsule (SIGBIC). The blue arrow shows the water-droplet signal.

The diagnostic MRI showed bilateral capsular contracture associated with implant shell permeability changes, described as a water-droplet signal. The MRI also showed signals of gel bleed described as intracapsular masses with late contrast enhancement, silicone induced granuloma of breast implant capsule (SIGBIC), Figures 1, 2. The patient underwent *en bloc* capsulectomy, followed by fat grafting breast reconstruction in December 2020.

The materials of the *en bloc* capsulectomy and wrist surgery were analyzed with standard H&E staining and an additional MORO staining was performed to detect silicone gel bleed and/or migration. The MORO staining is described in detail in gel bleed and rupture (5).

In the H&E staining, silicones can be recognized as glassy, non-birefringent droplets in vacuoles within the tissue or intracellular in macrophages. MORO staining showed a positive result for silicone in the capsules and synovia.

**Figure 3:**
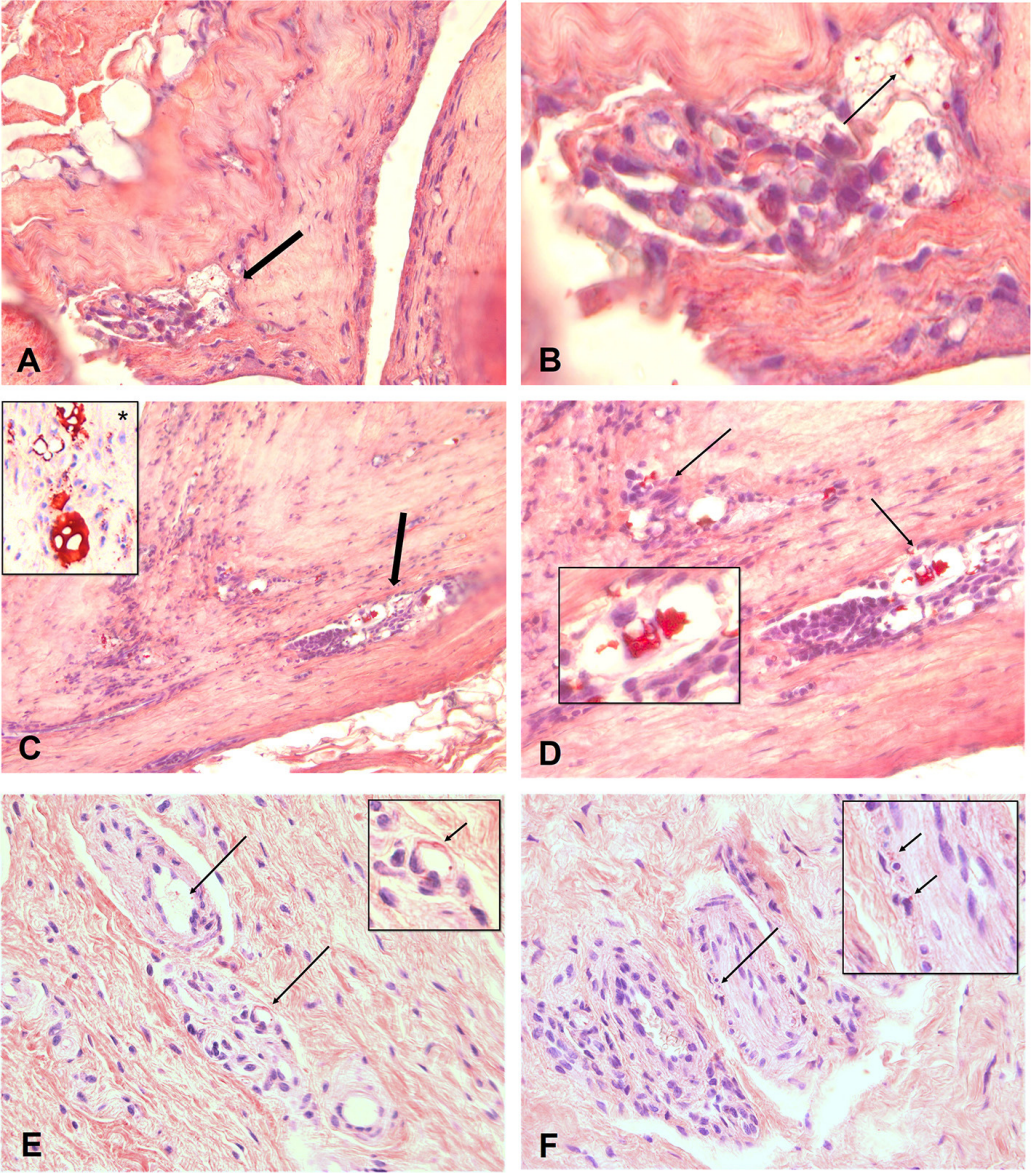
**(A–F)** Show 4 μm paraffin slides stained with the MORO. An overview of capsule from patient with focal throughout the capsule, spots who are positive. A magnification of designated area's shown in B. Clearly to distinguish are the vacuoles filled with red coloured silicone fragments. During degradation these fragments become smaller. Bigger fragments are showed in **(C,D)** with a positive control visible in inset *. This gradual dispersion can be found throughout the capsule. Degradation of the silicone can be seen everywhere and eventually the silicone molecules will migrate into the body and can reach every spot. **(E)** Shows a silicone spot in the synovia of the arm. Small spots are scatterd true the interstitium entering the bloodvessels (arrow). These vacuoles and especially the small granules can also be found in small nervebundles, **(F)**. Original microscopic magnification; **(A,C)** 100X, **(B, D–F)** 400x.

Figures 3A–F show a 4 μm paraffin slide stained with MORO. Throughout the capsule, there are large foci, which are strongly positive. The vacuoles filled with red-colored silicone fragments which become smaller during degradation are clearly distinguished. Larger fragments are shown in Figures 3C,D with a positive control visible, inset ^*^. This gradual dispersion of silicones can be found throughout the capsule.

Figure 3E shows an area with smaller granular in the synovia of the arm, scattered through the interstitium and clearly visible in the wall of a blood vessel (arrow). These vacuoles, and especially the small granules, can also be found in small nerve bundles (Figure 3F).

## Discussion

Silicone is an artificial molecule that is not present in the environment. The first attempts to apply silicones subcutaneously began as early as the late 1940s, and at that time, studies by Rowe and co-workers suggested that silicones were physiologically inert and presented no health risks.

Silicone in SBIs is usually straight chains of polydimethylsiloxanes (PDMS). PDMS fluids come in various viscosities from water-like liquids to non-pourable fluids, and all of them are essentially water-insoluble (hydrophobic). Their contents were mixtures of various sized polymer compounds, many of which are smaller than the pores of the shell (6) and consequently bleeding occurrs (7). The latest generation of SBIs consists of a high cohesive gel but also in these latest types, gel bleed still occurs and will increase over time, due to biodegradation of the cohesive gel and the implant shell (8–10).

The result of silicones bleeding from the SBI is that they are able to trigger an immune response. Furthermore, due to biodegradation by enzymes and hydrolysis, the silicones are degraded to cyclic siloxanes leading to toxicity and further rupture (9, 11, 12).

The increase in the number of silicones in tissues is often associated with an increase in the numbers of macrophages, fibroblasts, giant cells, and contractile myofibroblast (13, 14), all of which are related with several symptoms of BII (15). Further, the surface and texture of silicone breast implants can impact the immune system (16). In our study, we have used MORO stain to show silicone migration in tissues–synovia, blood vessels, and nerve bundles, *via* the specific staining of silicone polymers (5).

We also describe the correlation of the MRI findings to histology, regardless of no evident intracapsular rupture. The MRI accurately showed gel bleed and therefore MRI could be used as a marker for gel bleed and silicone migration. When silicone-induced granuloma of breast implant capsule (SIGBIC) is present, the nonspecific symptoms reported by patients should be valorized by the clinician using MRI to further diagnose BII.

In many patients who have complaints about their breast implants, removal (explanting) of the SBI leads to a considerable decline in complaints in a lot of patients, a strong indicator of a causative relation (17, 18).

Accordingly, in our patient, there was a significant improvement in reported symptoms after explantation, such as muscle contractures, arthralgias, shortness of breath, urticaria, fatigue, and arthritis. However, at present (2021), pain and paresthesia persist in the wrists, especially on the right, as well as the persistence of immunological markers and Raynaud Phenomenon.

Silicone breast implant toxicity is a cause for concern toward the unborn child because silicone molecules can pass the placenta and miscarriages occur (15), supported by increasing scientific evidence (19). It is of great importance there is a more intensified discussion for the diagnosis of BII, silicone gel bleed, and migration in the medical community.

## Data Availability Statement

The original contributions presented in the study are included in the article/supplementary material, further inquiries can be directed to the corresponding author.

## Ethics Statement

Ethical review and approval was not required for the study on human participants in accordance with the local legislation and institutional requirements. The patients/participants provided their written informed consent to participate in this study. Written informed consent was obtained from the individual for the publication of any potentially identifiable images or data included in this article.

## Author Contributions

All authors listed have made a substantial, direct, and intellectual contribution to the work and approved it for publication.

## Conflict of Interest

The authors declare that the research was conducted in the absence of any commercial or financial relationships that could be construed as a potential conflict of interest.

## Publisher's Note

All claims expressed in this article are solely those of the authors and do not necessarily represent those of their affiliated organizations, or those of the publisher, the editors and the reviewers. Any product that may be evaluated in this article, or claim that may be made by its manufacturer, is not guaranteed or endorsed by the publisher.
